# Plymouth Isolation Hospital

**Published:** 1898-10-01

**Authors:** 


### Plymouth Isolation Hospital.

A special meeting of the sub-committee of the Plymouth Town Council which has the management of the Isolation Hospital has been held to investigate the charges brought against that hospital in a report lately adopted by the Plymouth Board of Guardians, and prepared by the Visiting Committee of that authority, a report on which we made certain comments last week. According to the report of this meeting which appears in the *Western Evening Herald*, and the accompanying reply of the committee, which "the town clerk was instructed to supply to the Press," the whole of the allegations were gone into most exhaustively, with the result of showing that "there is not the slightest foundation for the charges reflecting on the management of the hospital." The statement drawn up by the committee says:—

"The rules of the institution with regard to the discharge of patients prevent the possibility of the discharge of patients in the dirty condition described in the report. In the cases mentioned in the report the sub-committee have received ample and abundant testimony that the rules were observed, and are satisfied that all patients named were properly treated, and were sent away in a clean and proper condition; and they have further been assured by Dr. Cooke, who was in charge during Dr. Williams' absence, and who is also the medical officer of the Plymouth Workhouse, that when he saw them in the workhouse on the day following their re-admission, the children referred to in the Guardians' report were clean and free from vermin."

It is added that the most important witness was Dr. Cooke, who absolutely denied that any of the children were sent away in a dirty or sore condition. On the contrary, the suggestion was made that when some of the children entered the hospital their heads were dirty, and their hair had to be cut by the barber.

When charges of this sort are floating about it is always, in our opinion, desirable that they should be publicly made and inquired into. Nothing can be more unsatisfactory than that vague rumours of bad management should fly about unrefuted, for, as we said last week, the proceedings within the walls of isolation hospitals are of necessity much more removed from the public eye than are those in other hospitals. It must, then, be a cause of much satisfaction to the Plymouth Corporation to find that the charges made by the Guardians against the hospital have been met in the manner that they have been. Now we wonder what *amende* or otherwise the Guardians will make.

